# Criterion validity of a Wechsler-III Scale Short Form in a sample of brazilian elderly

**DOI:** 10.1590/S1980-57642010DN40300009

**Published:** 2010

**Authors:** Eliane Ferreira Carvalho Banhato, Isabel Cristina Gonçalves Leite, Danielle Viveiros Guedes, Alfredo Chaoubah

**Affiliations:** 1 Brazilian Health Graduate Program, Federal University of Juiz de Fora, Psychology Professor at the Center for Higher Education of Juiz de Fora, Juiz de Fora MG, Brazil.; 2 Department of Collective Health, Federal University of Juiz de Fora, Juiz de Fora MG, Brazil.; 3 Psychology Graduate Program, Federal University of Juiz de Fora, Juiz de Fora MG, Brazil.; 4 Department of Statistics. Federal University of Juiz de Fora, Juiz de Fora MG, Brazil.

**Keywords:** Wechsler Intelligence Adult Scale, short forms, aged, cognitive impairment

## Abstract

Although a normative process, changes in cognitive functioning vary among older adults. The differential diagnosis between normal and pathological aging must be made early using psychometrically adequate measures.

**Objectives:**

To assess the evidence of criterion validity of a Short Form (SF) of the Wechsler-III Scale containing eight subtests (SF8) by determining its sensitivity, specificity, positive and negative predictive values and cut-off points for Brazilian elderly from different age groups.

**Methods:**

168 individuals, aged 60 years or above, living in the community or in an institution, were assigned to case and control groups, and investigated according to age range. Measures included a sociodemographic questionnaire, the Mini-Mental State Examination (MMSE), Verbal Fluency Test, Clock-Drawing Test and the SF8.

**Results:**

More than two thirds of the sample was women (73.8%), mean age was 74.5 years (SD=8.9), mean education was 6.2 years (SD=4.8) and 40.5% were widows/widowers. In the total sample, the best cut-off point for the SF8 was 142 while cut offs among individuals aged 60 to 69 years, 70 to 79 years, and more than 80 years were 160, 129 and 129, respectively.

**Conclusions:**

The results demonstrated the importance of different cut-off points for different age ranges. Sensitivity and specificity values of the SF8 were sufficiently high to warrant the use of the SF8 as an instrument to identify cognitive impairment in the elderly.

## Abstract (pt)

Embora normativas, as alterações no funcionamento cognitivo variam entre os idosos. O diagnóstico diferencial entre envelhecimento normal e patológico deve ser obtido precocemente, utilizando instrumentos psicometricamente adequados.

**Objetivos:**

Verificar evidências de validade de critério de uma Forma Abreviada da escala Wechsler-III com oito subtestes (FA8) pela determinação de sua sensibilidade, especificidade, valores preditivos positivo e negativo e pontos de corte para idosos brasileiros de diferentes faixas etárias.

**Métodos:**

168 indivíduos de 60 ou mais anos, residentes na comunidade ou institucionalizados, foram divididos em grupos caso e controle de mesma dimensão e avaliados de acordo com a faixa etária. Os instrumentos incluíram um questionário sociodemográfico, o MEEM, o Teste de Fluência Verbal, o Teste do Desenho do Relógio e a FA8.

**Resultados:**

Mais de dois terços da amostra foram compostos por mulheres (73.8%), a média etária foi de 74,5 anos (DP=8,9), a média de escolaridade foi de 6,2 anos (DP=4,8) e 40,5% eram viúvos. Na amostra total, o melhor ponto de corte para a FA8 foi 142; entre os idosos de 60 a 69 anos, de 70 a 79 anos e de 80 anos ou mais foram 160, 129 e 129, respectivamente.

**Conclusões:**

Os resultados demonstraram a importância de pontos de corte diferenciados entre as faixas etárias. Os valores de sensibilidade e especificidade da FA8 foram suficientemente altos, permitindo a utilização da FA8 como ferramenta na identificação de declínio cognitivo em idosos.

In Brazil, individuals over 60 years of age represent 8.6% of the total population. In 2025, the country is expected to rank sixth worldwide in the number of elderly citizens.^32^ Along with this demographic transition, there is an epidemiologic transition, with an increase in the prevalence of chronic-degenerative diseases, primarily different types of dementia.^7^

Cognitive change is normative in later life, although does not affect all elderly in the same way.^17^ The differential diagnosis between normal and pathologic aging must be made early, so that appropriate interventions, monitoring and treatment can be more effective.

In mental health research, specifically in epidemiologic studies, the efficacy of early diagnosis of pathologies and impairments has been increasingly acknowledged. Thus, cognitive screening and detailed assessment, including complex neuropsychological measures, are increasingly relevant.^25^

Among the several screening tests in available, the Mini-Mental State Examination (MMSE),^13^ Verbal Fluency Test^15^ and Clock-Drawing Test^28^ are the most frequently used. The MMSE has been widely used for global, quick and simple screening of human cognition. Considering different MMSE versions and scoring systems, a consensus statement was issued by the Scientific Department of Cognitive and Aging Neurology of the Brazilian Academy of Neurology.^19^ The consensus recommended the use of a single version in the country thereby standardizing the use of MMSE. The recommended version was that proposed by Brucki et al.,^6^ whose applicability was found satisfactory among older adults in different settings including hospitals, outpatient and community environments. This version does not suggest the use of cut-off points, but a classification from medians and means obtained by the individuals, taking into account the education level of subjects. Thus, we perceive that a stricter criterion in using the MMSE scale helps to reduce the influence bias of education on the individual performance in the MMSE.

For specific investigation of cognitive functions, the third edition of the *Wechsler Adult Intelligence Scale* (WAIS-III)^33^ is a battery which has been used to measure the intellectual coefficient in a large number of studies with adults.^2,11,32,35^ The WAIS-III consists of fourteen sub-tests with an increasing level of difficulty, divided into four factorial indexes (oral comprehension, perceptual organization, working memory and processing speed). Even though the WAIS-III has been adapted and validated in Brazil,^18^ few studies are currently using this test in the elderly population. Among possible reasons explaining low utilization of this test we identify:

1. Length of time needed to complete the test;
2. Fatigue during the evaluation;
3. Attention dispersion; and
4. Loss of motivation.^11,21,27^

An alternative for its use in elders are the WAIS-III shorter forms (SF). Christensen, Girard and Bagby^8^ validated a shorter version with eight sub-tests (SF8) in a psychiatric sample and obtained a multi-factorial intelligence assessment tool with shorter application time. In Brazil, an investigation of the psychometric performance of different SF (with 2, 4, 7 and 8 subtests) identified high precision and validity of the SF8, concluding that the latter can safely substitute the full WAIS-III.^9^ Unlike other flexible instruments, because the SF8 is composed of sub-tests it is even more flexible allowing specific investigation of several cognitive domains.

However, both the WAIS-III scale and its SF8 shorter version do not have cut-off points limiting their usability. Having a cut-off point in neuropsychological evaluations is crucial for screening cognitive impairment as well as comparing performance across different cognitive domains.

## Objectives

The objective of this study was to assess the criterion validity of the SF8 in the identification of cognitive impairment by determining the scale’s sensitivity, specificity, positive and negative predictive values, and cut-off points for Brazilian elderly of different age groups.

## Methods

### Subjects

This study enrolled 168 subjects of both genders, aged 60 years or above, from the community (n=142; 84.5%) or nursing homes (n=26; 15.5%). Exclusion criteria included severe cognitive deficit, previously diagnosed loss of visual and/or hearing ability, motor changes, and neurologic or psychiatric problems.

The study was authorized (authorization number 205/2007) by the Research Ethics Committee of the Federal University of Juiz de Fora. All participants signed an informed consent form approved by the committee.

### Procedures

A cross-sectional study was conducted in a medium-sized city in the Southeastern region of Brazil (State of Minas Gerais) which has 10.6% of elderly people in the population.^14^ Community-dwelling subjects were either recruited from a previous study on the prevalence of dementia in the elderly in the city of Juiz de Fora^3^ (n=66; 46.5%) or by referral from health professionals (n=76; 53.5%). Older adults in nursing homes were selected by the staff.

Trained research staff (psychologists and psychology students) described the study, reviewed the informed consent and invited older adults to participate. After agreeing to take part in the study, participants signed the informed consent. Research staff scheduled participants’ assessment for a later date.

The criterion for sample selection formed case and control groups based on MMSE performance and the Neuropsychological Evaluation applied by the specialist. Thus, we conducted an initial evaluation using the MMSE screening test proposed by Brucki et al.^6^ to classify older adults as cognitively normal (control group) or impaired (case group). In the evaluation proposed by these authors, older adults were categorized according to age range (60 to 69 years; 70 to 79 years; 80 years or above), with their scores being assessed by educational levels. Although the authors did not define cut-off points for the MMSE, they recommended comparisons of the scores obtained with 20, 25, 27, 28 and 29 medians for the illiterate, those with 1 to 4 years of schooling, 5 to 8 years of schooling, 9 to 11 years of schooling, and those with over 11 years of schooling, respectively. Subsequently, trained research staff assessed socio-demographic data, the Verbal Fluency Test, Clock-Drawing Test and SF8. This process was performed at the patient’s home and took an average of 40 minutes.

For the SF8 assessment - composed of the Vocabulary, Similarities, Arithmetic, Digit Span, Picture Completion, Matrix Reasoning, Digit Symbol-Coding and Symbol Search subtests^9,18^ - we should point out that raw scores have been taken into consideration. The proportion/ratio technique (proratio) has been used in order to calculate the total score of the SF8, considering the sum of the scores of the subtests is increased by a factor that subtracts the subtests omitted in the complete scale [FA8=1.5 × (Vocabulary + Similarities) + 1.5 × (Picture Completion + Matrix Reasoning) + 1.5 × (Arithmetic + Digit Span) + 1 × (Digit Symbol-Coding + Symbol Search)].^9^

### Statistical methods

The data were analyzed with the SPSS 15.0 statistical program. Measures of central tendency for the socio-demographic variables, association measures (chi-squared) and comparison of the mean values (t test) were obtained. Pearson’s coefficient was used to examine correlations among continuous variables. In assessing the SF8 measure, sensitivity and specificity were analyzed through the Receiver Operator Characteristic (ROC) curve. Positive and negative predictive values were subsequently calculated. We adopted the .05 and 0.01 levels to establish significance.

## Results

### Sociodemographic characteristics of the sample

Of the 168 subjects assessed, the majority were women (χ^2^=38.09; p<0.001). Mean age was 74.5 years (SD=8.9), with a range from 60 to 98 years. When the elderly were considered by age range, no statistically significant difference in group size was found (χ^2^=0.25; p=0.88). Average education was 6.2 years (SD=4.8), with most participants having completed between 1 and 4 years of education (χ^2^=79.92; p<0.001). Regarding marital status, most subjects were widows or widowers (χ^2^=48.78; p<0.001)(Table 1).

**Table 1:** Sociodemographic characteristics of the total sample.

		n	%
Gender	Male Female	44 124	26.2 73.8
Age	60-69 years 70-79 years ≥80 years	55 59 54	32.7 35.1 32.1
Schooling	Illiterate 1-4 years 5-8 years 9-11 years > 11 years	14 79 28 22 25	8.3 47.0 16.7 13.1 14.9
Marital status	Married / stable relationship Divorced Widowed Single	60 16 68 23	35.9 9.6 40.7 13.8

### Cognitive performance

The SF8 score ranged from 12.00 to 361.50 (M=147.76; SD=77.29). Pearson’s coefficient identified high correlation between SF8 and education (r=0.73; p<0.001) and there was also a negative correlation between SF8 and age (r= –0.29; p<0.001).

Using the MMSE with the criteria proposed by Brucki et al.^7^ we identified 88 elderly (52.4%) with cognitive impairment. No significant difference was found between case and control groups (χ^2^=0.38; p=0.54). When cognitive impairment was taken into account, no significant difference in gender (χ^2^=0.13; p=0.71), age (t=1.25; p=0.21) or marital status (χ^2^=6.95; p=0.07) was found between intervention and control groups. In contrast, a difference was found between elderly with and without impairment in terms of schooling (t= –3.04; p<0.05) where those with cognitive impairment had less schooling.

### Validation of the SF8 scale

Pearson’s correlations were used to assess the association between SF8 and its subtests with Verbal Fluency Test, Clock-Drawing Test and MMSE. Correlations ranged from moderate to strong (0.52-0.80) in the expected direction as presented in Table 2.

**Table 2:** Correlation matrix between SF8, its subtests and Verbal Fluency Test, Clock Drawing Test and MMSE

	Verbal Fluency Test	Clock-Drawing Test	MMSE
SF8	0.69**	0.76**	0.80**
Vocabulary	0.63**	0.64**	0.77**
Similarities	0.57**	0.66**	0.72**
Arithmetic	0.62**	0.64**	0.68**
Digit Span	0.52**	0.54**	0.63**
Picture Completion	0.54**	0.68**	0.67**
Matrix Reasoning	0.53**	0.60**	0.60**
Digit Symbol-Coding	0.62**	0.73**	0.66**
Symbol Search	0.63**	0.68**	0.66**

** p<0.01

Different cut-off points were assessed using the proposed criteria for cognitive impairment, sensitivity, specificity, positive and negative predictive values, and the index of misclassification.

Using the total sample and its stratification according to the age ranges, cut-off points for the SF8 and each one of its subtests were identified. Table 3 presents the selected cut-off points, sensitivity, specificity and accuracy of all subtests included in the SF8.

**Table 3:** Description of the cut-off points for the SF8 and its subtests.

	Scale and subtests	Cut-off point	Sensitivity (%)	Specificity (%)	Area under the curve	Standarderror	Confidence interval (CI95%)
60-69 years	SF8 Vocabulary Similarities Arithmetic Digit span Picture completion Matrix reasoning Digit symbol-coding Symbol search	160 31 16 9 12 8 7 23 10	71.4 71.4 64.3 71.4 67.9 75.0 64.3 64.3 78.6	70.4 59.3 63.0 77.8 81.5 74.1 74.1 70.4 66.7	0.77 0.70 0.71 0.84 0.75 0.78 0.74 0.76 0.75	0.06 0.07 0.07 0.05 0.07 0.06 0.07 0.07 0.07	0.64-0.90 0.56-0.84 0.57-0.85 0.73-0.95 0.62-0.88 0.66-0.90 0.61-0.88 0.62-0.89 0.61-0.88
70-79 years	SF8 Vocabulary Similarities Arithmetic Digit span Picture completion Matrix reasoning Digit symbol-coding Symbol search	129 28 13 7 10 8 6 19 10	85.7 78.6 71.4 75.0 75.0 71.4 64.3 75.0 71.4	80.6 77.4 80.6 67.7 74.2 77.4 71.0 80.6 80.6	0.84 0.82 0.79 0.77 0.81 0.78 0.78 0.79 0.83	0.05 0.06 0.06 0.06 0.05 0.06 0.06 0.06 0.05	0.74-0.94 0.71-0.93 0.67-0.91 0.65-0.90 0.71-0.92 0.67-0.90 0.66-0.89 0.67-0.91 0.72-0.93
≥80 years	SF8 Vocabulary Similarities Arithmetic Digit span Picture completion Matrix reasoning Digit symbol-coding Symbol search	129 30 12 6 9 5 5 14 6	83.3 83.3 83.3 83.3 75.0 70.8 83.3 70.8 75.0	80.0 80.0 73.3 66.7 70.0 73.3 66.7 73.3 73.3	0.87 0.89 0.83 0.73 0.77 0.81 0.81 0.79 0.80	0.05 0.05 0.06 0.07 0.06 0.06 0.06 0.06 0.06	0.78-0.97 0.80-0.98 0.72-0.94 0.59-0.87 0.65-0.90 0.69-0.92 0.69-0.92 0.67-0.91 0.68-0.92
Total sample	SF8 Vocabulary Similarities Arithmetic Digit span Picture Completion Matrix Reasoning Digit Symbol-Coding Symbol Search	142 31 14 8 10 6 6 18 9	80.0 71.3 72.5 65.0 71.3 71.3 65.0 68.8 72.5	77.3 75.0 72.7 78.4 70.5 72.7 72.7 70.5 72.9	0.83 0.82 0.78 0.78 0.78 0.79 0.77 0.77 0.79	0.03 0.03 0.04 0.04 0.04 0.03 0.04 0.04 0.03	0.77-0.89 0.75-0.88 0.71-0.85 0.71-0.85 0.71-0.85 0.72-0.86 0.70-0.84 0.70-0.85 0.72-0.85

### ROC analysis

ROC curve analysis was carried out to obtain the discriminatory profile of the SF8 scale. The ROC consists of a mathematical tool whose aim is to establish the several possible cut-off points, as well as the indexes of true positive (sensitivity) and false-positive (1-specificity) for all cut-off points. The area under the ROC curve (AUC) is a global index of the discriminative precision of the curve. Table 3 shows the AUC values of the SF8 for the different age ranges as well as for the subtests.

Table 4 shows the proportion of true positives among all subjects with scores lower than the cut-off point (positive predictive value - PPV) and of the true negatives among all subjects with scores higher than the cut-off point (negative predictive value - NPV).

**Table 4:** Predictive values of the SF8 subtests.

	Positive predictive value (%)					Negative predictive value (%)			
	**60-69 years**	**70-79 years**	≥ **80 years**	**Total sample **		**60-69 years**	**70-79 years**	≥ **80 years**	**Total sample **
SF8	70.0	80.6	80.0	77.3		71.4	85.7	83.3	80.0
Vocabulary	59.2	77.4	80.0	75.0		71.4	78.6	83.3	71.2
Similarities	63.0	80.6	73.3	72.7		64.3	71.4	83.3	72.5
Arithmetic	77.7	67.7	66.7	78.4		71.4	75.0	83.3	65.0
Digit span	77.7	71.0	70.0	70.0		67.9	75.0	75.0	71.2
Picture completion	74.1	77.4	73.3	72.7		75.0	71.4	70.8	71.2
Matrix reasoning	74.1	71.0	66.6	72.7		64.3	60.7	83.3	65.0
Digit symbol-coding	70.4	77.4	73.3	70.4		64.3	75.0	70.8	68.7
Symbol search	69.0	80.6	73.3	72.7		78.6	71.4	75.0	72.5

## Discussion

The importance of improving the discriminatory aspects of existing neuropsychological assessment tools among older adults has increased substantially, due to changes in the demographic and epidemiologic profiles, especially given the high prevalence of degenerative diseases such as the dementias.^16,22,25^ The literature highlights the importance of the WAIS-III scale as the gold standard for cognitive assessment.^1,11,32,35^ Identification of cut-off points for this scale and assessment of its short form among the elderly is innovative and has clinical, academic and epidemiologic relevance.

The moderate to strong correlations between the tests used showed that they all assess similar constructs, that is, cognition. Our results point to a negative correlation of age and score on the SF8. This result is in accordance with the literature as regards decline in cognitive skills, even in normal aging, especially concerning the performance of rapid, attention, concentration and inductive reasoning-related tasks.^28,31^

This study also identified different cut-off points for elderly from different age ranges (young elderly, mid-elderly and old-elderly), allowing a comparison among more homogeneous age groups. This reveals aging as a heterogeneous process in which different profiles of cognitive development occur. When considering the cut-off points for each age group, it was possible to determine that levels generally decrease inversely proportional to age. On the other hand, the same cut-off point was found for the old-elderly (age greater than or equal to 80 years) and for the mid-elderly (70 to 79 years) on the SF8. Contrary to expectations, the Vocabulary subtest cut-off point was higher for the old-elderly than the mid-elderly, a finding that could be explained by the fact that the crystallized intelligence evaluated by this subtest can be maintained in the aging process.

Variables concerning the present environment, life history, individual action and subjective assessments are thought to provide multiple old-age realities, within objective and subjective health and physical, social and psychological well-being conditions.^4,5,12^ This leads us to consider that our study sample might have been influenced by a cohort effect whereby this group of old-older adults may have different variables compared to the middle-old.

The strong positive correlation identified between the SF8 and education is in accordance with the literature review, which shows the influence of this variable on cognitive performance measured by both screening instruments and flexible batteries.^20,26,30^ However, although education interferes with the performance on the MMSE, this scale is recommended by several studies.^5,10,23-26^

The study of the cut-off points of the SF8 subtests prioritized the scores with higher specificity, since it is a scale used for neuropsychological assessments aimed at differential diagnosis. In general, considering the age ranges and total sample, diagnostic accuracy was psychometrically satisfactory for the cut-off points used in the subtests (AUC between 0.70 and 0.89). Although these results show the ability of the scale to discriminate cases and non-cases, it must be emphasized that, used alone, it cannot reach a differential diagnosis. However, its use combined with other scales – such as functional capacity evaluations - can significantly improve the diagnosis.

Our results showed that the SF8 has the advantage of being quicker to complete, in comparison with the full scale, while providing comfort for and greater involvement of the participants. The use of crude scores allowed the identification of the cut-off points and enabled a more practical scale correction compared to the traditional approach which considers weighted scores (average equals ten and standard deviation equals three).

In addition, by using different reference scores for elderly from different age ranges, it is possible to introduce a new criterion pattern and standardized evaluations. Thus, the scale can be used to categorize the subjects according to their cognitive capacity.

Furthermore, sensitivity and specificity were sufficiently high, making the test a useful neuropsychological instrument to discriminate subjects with and without cognitive impairment. However, these results must be analyzed with caution, since the prevalence of cognitive impairment in this sub-sample (pre-test probability) cannot be extrapolated to the general population. In the present study, sample selection from a population-based triage and clinical indications may have produced a selection bias. Consequently, the prevalence of subjects without cognitive impairment may have been higher than that found in the general population.

Because there are no similar investigations in the area our results cannot be compared, representing a limitation of this study. Thus, case-control studies involving larger samples and which take into account the prevalence of cognitive impairment in the population are necessary to test these cut-off points and find additional evidence of the validity of the SF8.
